# The role of genetic factors in pediatric myelodysplastic syndromes with different outcomes

**DOI:** 10.1186/s12887-023-04492-2

**Published:** 2024-01-08

**Authors:** Ying Li, Li Cheng, Yun Peng, Lin Wang, Wenzhi Zhang, Yuhong Yin, Jing Zhang, Xiaoyan Wu

**Affiliations:** Department of Pediatrics, Union Hospital, Tongji Medical College, Huazhong University of Science and Technology, Wuhan, 430022 China

**Keywords:** Pediatric MDS, Germline mutations, Somatic mutations, Outcomes, Disease characteristics

## Abstract

**Background:**

Pediatric myelodysplastic syndromes (MDS) are rare disorders with an unrevealed pathogenesis. Our aim is to explore the role of genetic factors in the pathogenesis of MDS in children with different outcomes and to discover the correlation between genetic features and clinical outcomes as well as disease characteristics.

**Methods:**

We conducted an analysis of archived genetic data from 26 patients diagnosed with pediatric MDS at our institution between 2015 and 2021, examining the association between different genetic characteristics and clinical manifestations as well as prognosis. Additionally, We presented three cases with distinct genetic background and outcomes as examples to elaborate the role of genetic factors in pediatric MDS with different prognoses.

**Results:**

Genetic variations were detected in 13 out of the 26 patients, including 8 patients with co-occurrence of somatic and germline mutations (CSGMs) and 5 patients with somatic mutations alone. Our analysis revealed that advanced MDS (4/8, 50% vs. 1/5, 20% and 4/11, 36.4%), PD (3/8, 37.5% vs. 1/5, 20% and 1/11 9.1%), and TD (6/8, 75% vs. 2/5, 40% and 2/11, 18.2%) were more common in patients with CSGMs than those with somatic mutations alone or without any mutations. We also found out in our study that 8 patients with CSGMs had evidently different clinical outcomes, and we presented 3 of them as examples for elaboration. Case 1 with germline and somatic mutations of unknown significance had a relatively slow disease course and a good prognosis. Case 2 with compound heterozygous germline SBDS variants and somatic mutations like del20q had a stable disease course and a reversed outcome. Case 3 with a germline GATA2 variant and somatic mutations including − 7 had a rapidly progressive disease course and a worst prognosis.

**Conclusion:**

Our findings indicate that genetic background of pediatric MDS is closely linked with disease characteristics as well as outcomes and that CSGMs may lead to disease progression. It should be emphasized that the interaction between certain germline variants and somatic mutations, such as SBDS and del20q, may result in hematopoietic stem cell adaptation (improved hematopoiesis) and reversed clinical outcomes, which can facilitate the development of targeted therapy.

**Supplementary Information:**

The online version contains supplementary material available at 10.1186/s12887-023-04492-2.

## Background

Myelodysplastic syndromes (MDS) are a heterogeneous group of clonal hematopoietic disorders characterized by peripheral cytopenia, ineffective hematopoiesis, morphologic dysplasia, and an increased risk of progression into acute myeloid leukemia (AML) [1]. MDS mostly occurs in adults older than 70 years and is rare in children. Furthermore, pediatric and adult MDS vary considerably in terms of morphological features, genetic alterations, pathophysiology and therapeutic goals. In children, MDS is often associated with inherited bone marrow failure syndromes (IBMFs), genetic predisposition syndromes, and previous exposures to cytotoxic agents [2–4]. The role of germline genetic predisposition in children with MDS has received increasing attention with the application of gene sequencing technologies in clinical practice. Germline syndromes caused by variants in GATA2, ETV6, RUNX1, SAMD9/SAMD9-L and SRP72 have been reported susceptible to MDS [3–5]. However, the mechanisms underlying how germline predisposition leads to MDS are not completely understood and may be may involve acquired somatic mutations under a germline genetic background [6, 7]. In addition, adult genetic studies of MDS have shown that molecular alterations are closely associated with clinical outcomes and disease characteristics [8, 9].

However, the impact of these molecular alterations on clinical features and prognosis of pediatric MDS remains a mystery. Thus, we conducted a study to summarize the genetic data of 26 pediatric MDS patients diagnosed at our institution between 2015 and 2021. We presented three cases of them with different outcomes as examples for elaborating the possible role of genetic factors in pediatric MDS pathogenesis and the correlation between genetic features and disease characteristics as well as outcomes.

## Method

### Patients and samples collection

Twenty-six patients who were diagnosed with pediatric MDS with available genetic data at our institution between 2015 and 2021 were enrolled. The diagnosis and classification of MDS followed the latest WHO pediatric classification system [10]. Clinical specimens, including bone marrow (BM) as the source for somatic DNA and saliva as the source for germline DNA, were collected upon confirmation of disease diagnosis. The sample were promptly sent to Kindstar Global Medical Laboratory (Wuhan, China) for further analysis within 12 h of collection.

### Observed items

We mainly observed the changes in peripheral blood cell counts, transfusion status, infection status, BM morphology and blast cell percentage, disease progression and survival in pediatric patients during the disease course. Disease manifestations consist of transfusion-dependency (TD) and transfusion independency (TID). According to the International Working Group (IWG) response criteria [11], we define disease evolution as hematological improvement (HI), stable disease (SD), or disease progression (PD). HI contains complete remission (CR) and partial response (PR). PD includes TD for platelet (PLT) or red blood cell (RBC), reduction in hemoglobin by 20 g/L, severe neutropenia (<0.5 × 10^9^/L), and increased BM blasts from baseline (less than 5% blasts: ≥50% increase in blasts to > 5% blasts; 5–10% blasts: ≥50% increase to > 10% blasts; 10–20% blasts: ≥50% increase to > 20% blasts; 20–30% blasts: ≥50% increase to > 30% blasts). SD refers to no HI and no PD. Clinical outcomes include survival and death [12, 13].

### Genetic testing techniques

A total of 22 patients were evaluated by gene panel sequencing and 4 patients by whole exome sequencing (WES). Gene panel sequencing contained 67 genes selected on the basis of their known or suspected involvement in the pathogenesis of myeloid cancers.(Supplemental Table 1). Genomic DNA (gDNA) was extracted using the QIAamp DNA Blood Mini Kit according to the manufacturer’s instructions. DNA was sheared on the Covaris focused ultrasonicator. All libraries were prepared using the KAPA HTP Library Preparation Kit according to the manufacturer’s instructions. Fragmented DNA was repaired, 3’ dA-tailed, ligated with Illumina adapters, size selected, amplified, and assessed using the Agilent 2100 Bioanalyzer. The libraries were then subjected to sequencing on an Illumina NextSeq 550 instrument.

For WES, extracted gDNA samples were interrupted using a covaris M220 sonicator. The DNA libraries were then prepared following manufacturer’s instructions with the SureSelectXT Human All Exon V6 Reagent Kit. Quality control was performed using the Agilent Bioanalyzer 2100 and the High Sensitivity DNA Analysis Kit before performing 150-bp paired-end sequencing on an Illumina NovaSeq 6000 Sequencer. The raw data were converted from bcl files to fastq format files by Illumina CASAVA1.8. The reads were compared to the GRCh37/hg19 human genome reference using BWA, samtools, picard, and GATK to remove repeated sequences and identify genetic variants. All identified variants were evaluated by browsing through databases, including NCBI dbSNP, OMIM, HGMD and NCBI ClinVar.

## Results

### Clinical patient characteristics

A total of 26 patients with documented genetic data were included in this study, comprising 16 males (61.5%) and 10 females (38.5%). The median age at the onset of hematologic abnormalities was 5.87 years (ranging from 0.25 to 13 years) Pancytopenia was the predominant presentation in the majority of patients (50%), with median peripheral hemoglobin (Hb) levels, platelet counts (PLTs), and absolute neutrophil counts (ANC) of 78 (44–125 g/l), 54 (10–263 × 10^9^/l), and 1.41 (0.13–5.08 × 10^9^/l), respectively. Among the 26 patients, 16 had refractory cytopenia of childhood (RCC), and 10 had advanced MDS (including RAEB and RAEB-t). Cytogenetic abnormalities were observed in 9 out of the 26 patients (34.6%), with monosomy 7 being the most common aberration. Detailed information is given in Table 1.

**Table 1:** Clinical characteristics of 26 patients

Characteristic	Value
**Sex, no./total (%)**	
Male	16 (61.5%)
Female	10 (38.5%)
**Age at diagnosis, [years, median (range)]**	5.87 (0.25−13)
**Diagnosis, no./total (%)**	
RCC	16 (61.5%)
Advanced MDS	10 (38.5%)
**Hematological characteristics**	
Cytopenia, 1 lineage,no./total (%)	3 (11.5%)
Cytopenias, 2 lineages, no./total (%)	10 (38.5%)
Pancytopenia, no./total (%)	13 (50%)
Hb level,median (range), < 100 g/l	78 (44–125)
PLT count,median (range), < 100 × 10^9^/L	54 (10–263)
NE count, median (range), < 1.5 × 10^9^/L	1.41 (0.13–5.08)
**Abnormal karyotype, no./total (%)**	9 (34.6%)
Monosomy 7	5 (19.2)
Complex karyotype	1 (3.9%)
Other	3 (11.5%)
**Gene mutation**	13(50%)

Abbreviations: RCC, refractory cytopenia of childhood; Hb, hemoglobin; PLT, Platelet; NE, Neutrophil

### Clinical characteristics and outcomes

**Fig. 1:**
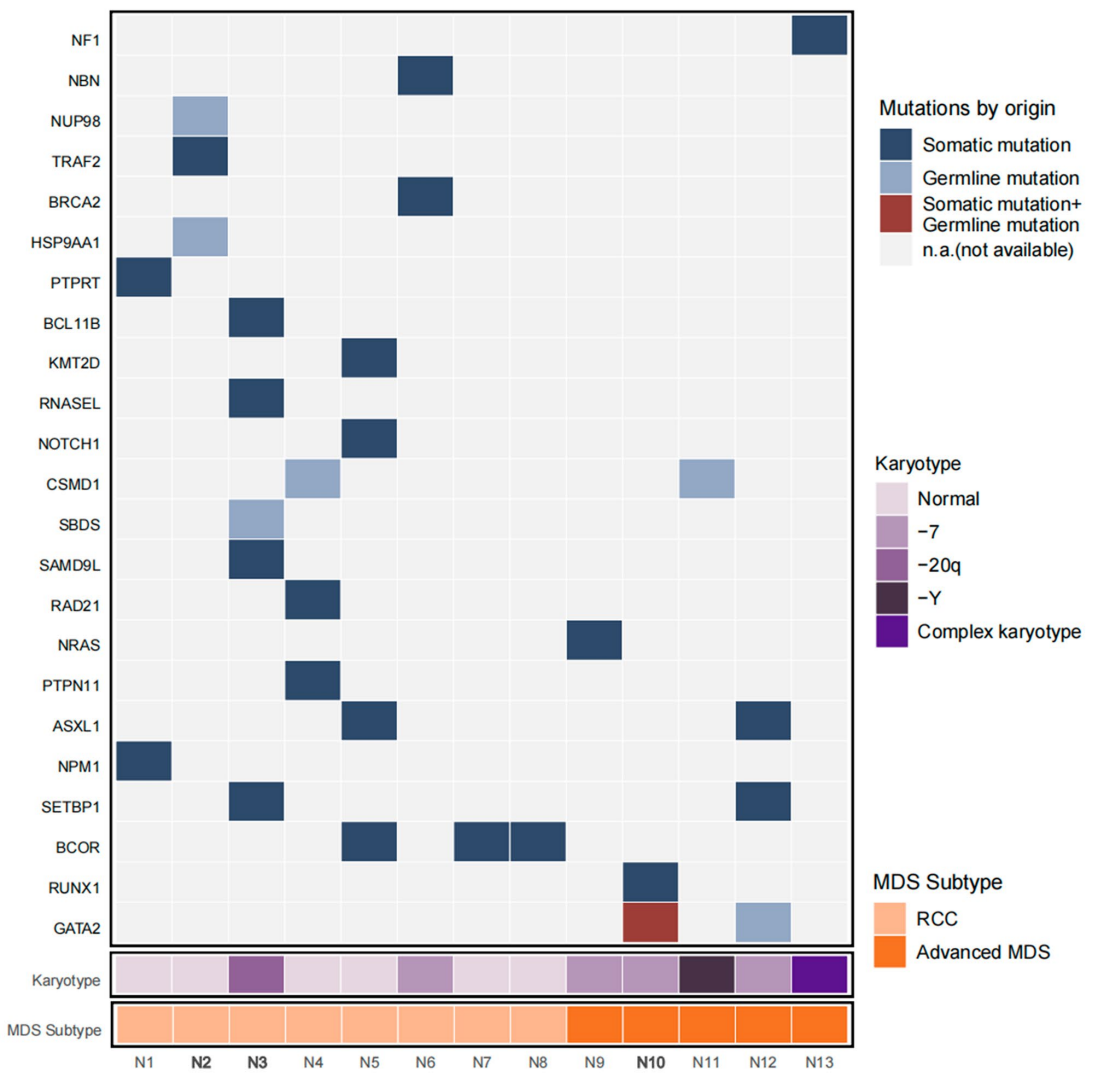
Overall mutational spectrum. Depicts the subtypes, mutation types and karyotypes (corresponding color coding as depicted in the legend) in all patients. RCC, Refractory cytopenia in children; MDS-EB, MDS with excess of blast; -7, monosomy 7; -20q, del20q; -Y, delY

**Fig. 2:**
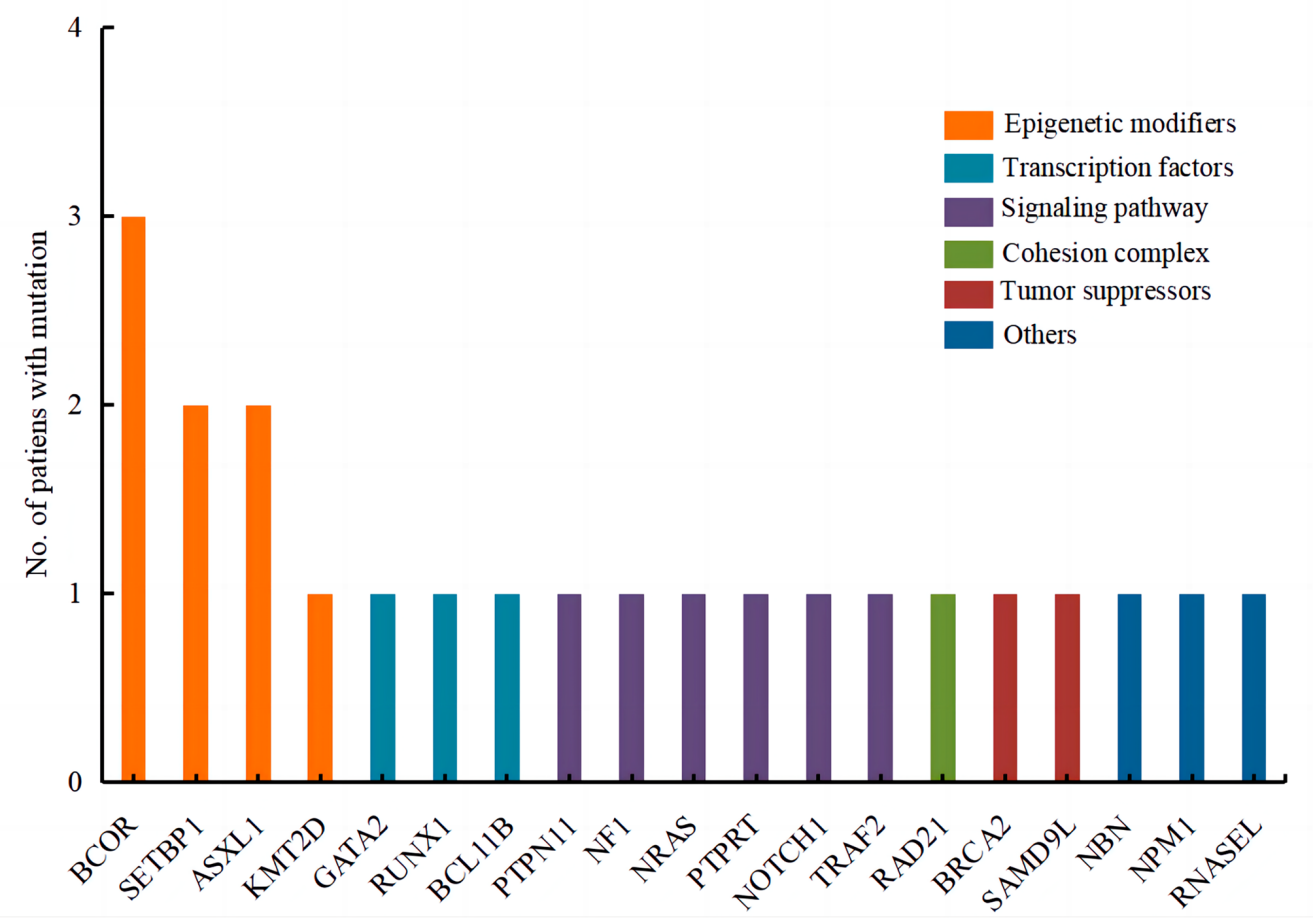
Analysis of gene mutations in 13 MDS patient

**Fig. 3:**
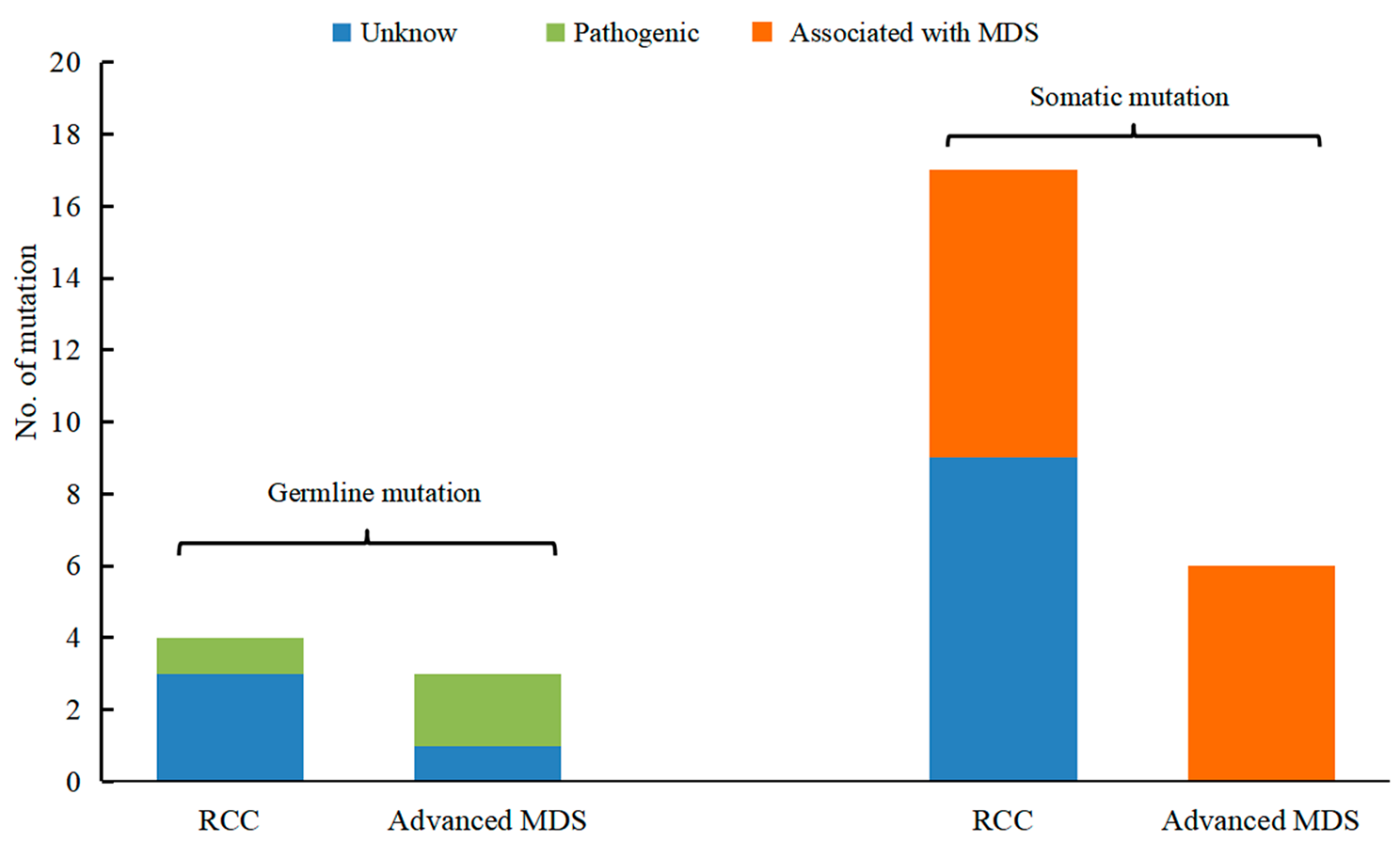
Comparision of gene mutations types in different subtypes of MDS

Of the 26 patients (4 using WES and 22 using targeted sequencing of 67 genes), 13 (50%) had genetic mutations, involving a total of 23 genes (Fig. 1). Germline validation confirmed the presence of germline mutations in 6 patients, including 1 with a compound heterozygous SBDS pathogenic variant, 2 with a GATA2 pathogenic variant, and 3 with germline mutations of undetermined significance (gVUS). Somatic mutations were detected in 12 patients, mainly involving transcription factors (GATA2, RUNX1), epigenetic modifications (BCOR, ASXL1, SETBP1), signaling transduction (PTPN11, NF1, NRAS), cohesion (RAD21), and other driver mutations (NPM1) (Fig. 2). Further analysis revealed that germline pathogenic variants (2/3 in advanced MDS and 1/4 in RCC) and MDS-related somatic mutations (6/6 in advanced MDS and 9/17 in RCC) were more prevalent in the advanced MDS group, and mutations of undetermined significance were mostly found in RCC (Fig. 3).

### Genetic characteristics and clinical outcomes

Of the 13 patients mentioned above, 8 patients had CSGMs and 5 patients contained somatic mutations only (cytogenetic abnormalities were further germline validated) (Table 2). Our results showed that advanced MDS (4/8, 50% vs. 1/5, 20% and 4/11, 36.4%), PD (3/8, 37.5% vs. 1/5, 20% and 1/11 9.1%) and TD (6/8, 75% vs. 2/5, 40% and 2/11, 18.2%) were more prevalent in patients with CSGMs than those with somatic mutations alone or without any mutations (Table 3). Of the 8 patients with CSGMs, 4 had RCC and the other 4 had advanced MDS. In 4 RCC patients with CSGMs, the somatic mutations were mostly of unknown significance, and the germline variants included 1 SBDS pathogenic variant, 1 monosomy 7, and 2 gVUS. Among these 4 patients, 2 had TD and 2 had TID. One of the TD cases (N3) adopted a watch and wait strategy due to HI, while the other TD case (N6) underwent allogeneic hematopoietic stem cell transplantation (HSCT). One of the TID cases (N2) performed HSCT for PD, and the other TID patient (N4) adopted a watch and wait strategy. All these 4 patients survived. Among the 4 advanced MDS patients with CSGMs, the somatic mutations were associated with MDS, and 2 of the germline variants were GATA2 pathogenic mutation, 1 was monosomy 7, and 1 was gVUS. All these 4 patients were TD, 1 patient with a germline GATA2 variant (N12) did not accept HSCT and died of PD. The remaining 3 patients received HSCT and 2 of them remained in CR, while the other patient with the germline GATA2 mutation (N10) died from severe post-transplantation infections (Table 2).

**Table 2:** Clinical characteristics and treatment outcomes of 13 patients with gene mutation

ID	subtypes	Sex/ Age(y)	TD at diagnosis	BM karyotype	Types of mutations	Treatment and disease evolution	Outcomes
**N1**	RCC	M/6	No	Normal	Somatic	CsA→SD	Survivlal
**N2**	RCC	F/5	No	Normal	Germline + somatic	Watch&wait→PD→CsA+HSCT→HI	Survival
**N3**	RCC	F/10	Yes	Del20q^**a**^	Germline + somatic	Watch&wait→HI	Survival
**N4**	RCC	M/0.58	No	Normal	Germline + somatic	CsA→SD	Survival
**N5**	RCC	M/9	No	Normal	Somatic	Watch & wait→SD	Survival
**N6**	RCC	M/0.3	Yes	45,XY,-7^**b**^	Somatic	HSCT→HI	Survival
**N7**	RCC	F/12	Yes	Normal	Somatic	HSCT→HI	Survival
**N8**	RCC	M/10	No	Normal	Somatic	CsA→HI	Survival
**N9**	MDS-EB	F/1	Yes	45,XX,-7^**b**^	Somatic	HSCT→HI	Survival
**N10**	MDS-EB	M/13	Yes	45,XY,-7^**a**^	Germline + somatic	PD→HSCT	Death
**N11**	MDS-EB	M/12	Yes	45,X,-Y^**a**^	Germline	HSCT→HI	Survival
**N12**	MDS-EB	F/10	Yes	45,XX,-7^**a**^	Germline + somatic	Support therapy (Give up HSCT)→PD	Death
**N13**	MDS-EB-t	M/2	Yes	Complex karyotype^**a**^	Somatic	Support therapy (Give up HSCT)→PD	Death

Abbreviations: F, female; M, male; TD, transfusion dependency; BM, bone marrow; y, years; m, months; SD, stable disease; PD, progressive disease; HI, hematological improvement; CsA, cyclosporine A; HSCT, hematopoietic stem cell transplantation; RCC, refractory cytopenia of childhood; MDS-EB, myelodysplastic syndromes with excess of blasts; MDS-EB-t, MDS-EB in transformation. a The chromosomal variant was verified as a somatic variant; b The chromosomal variant was verified as a germline variant

**Table 3:** Comparison of baseline characteristics and treatment of patients witht different genetic backgrounds

Characteristic	CSGMs (n = 8)	Only somatic mutation (n = 5)	Non-mutation* (n = 11)
**Age at diagnosis**, **[years, median (range)]**	6.37 (0.25−13)	7.8 (2–12)	4.8(0.75−11)
**Diagnosis, no./total (%)**			
RCC	4 (50%)	4 (80%)	7 (63.6%)
Advanced MDS	4 (50%)	1 (20%)	4 (36.4%)
**Hematological characteristics**			
Hb level,median (range)	69 (44–101)	82 (54–100)	83 (46–125)
PLT count,median (range)	53 (13–165)	45 (22–80)	62 (10–263)
NE count, median (range)	1.6 (0.39–5.08)	1.7 (0.83–2.82)	0.98 (0.13–1.95)
TD at diagnosis	6 (75%)	2 (40%)	2 (18.2%)
**Treatment at diagnosis, no./total (%)**			
Watch&wait / Support therapy	3 (50%)	2 (40%)	5 (45.4%)_
IST (CsA)	1 (12.5%)	2 (40%)	4 (36.4%)
HSCT	4 (50%)	1 (20%)	2 (18.2%)
**Response, no./total (%)**			
Hematological improvement(HI)	4 (50%)	2 (40%)	5 (45.4%)
Stable disease (SD)	1(12.5%)	2 (40%)	5 (45.4%)
Disease progression (PD)	3 (37.5%)	1 (20%)	1 (9.1%)
**Outcomes, no./total (%)**			
Survivl	6 (75%)	4 (80%)	10 (90.9%)
Death	2 (25%)	1 (20%)	1 (9.1%)

Abbreviations: CSGMs, co-occurrence of somatic and germline mutations; RCC, refractory cytopenia of childhood; Hb, hemoglobin; PLT, Platelet; NE, Neutrophil; TD, transfusion dependency; IST, immunosuppressive therapy; CsA, cyclosporine A; HSCT, hematopoietic stem cell transplantation; *Two patients with karyotypic abnormalities did not perform germline validation

### Detailed cases analysis

Case 1 (N2) was a 5-year-old girl who came to our hospital because of pancytopenia lasting for 3 months. Blood cell counts showed ANC of 0.68 × 10^9^/L, PLTs of 23 × 10^9^/L, and Hb levels of 10.1 g/dL. Initially, the diagnosis was unclear due to the lack of typical morphological and cytogenetic abnormalities. Regular BM aspiration was performed after a 2-year follow-up, revealing a hypocellular BM with granulocytic and erythroid dysplasia, leading to the diagnosis of RCC. With an ANC>0.5 × 10^9^/L and an unsupported PLT or red blood cells (RBC), a watch-and-wait strategy was used. After 4.5 years of follow-up, the child became TD for PLTs and the result of a repeat BM aspirate was consistent with RCC. WES detected germline NUP98 and HSP90AA1 variants of unknown significance, as well as a somatic TRAF2 mutation. A search for a suitable unrelated donor (UD) was then initiated due to the lack of an HLA-identical sibling. While awaiting a suitable UD, the child was treated with cyclosporine A (CsA), but the hematological parameters did not improve significantly. Fortunately, HSCT was performed immediately after a matched UD (in 9/10 HLA loci) was found. Two years after the HSCT, the child is still alive and in CR of her disease.

Case 2 (N3) involved a 10-year-old girl who was admitted to our hospital with intermittent fever and cough for 5 days. Her mother was previously diagnosed with depression. Physical examination showed evident growth retardation (weight 28 kg, height 110 cm; height < 3rd percentile for age). Routine blood tests revealed ANC at 1.4 × 10^9^/L, PLTs at 54 × 10^9^ /L and Hb at 8.0 g/dl. BM aspirate indicated a hypercellular BM with mild hematopoietic abnormalities, and cytogenetic analysis of the marrow cells suggested del20q in 18 metaphases analyzed (verified as somatic origin). Additionally, WES analysis identified 2 compound heterozygous mutations in the SBDS gene: c.183_184delTAinsCT and c.258 + 2 T > C. Both of these mutations have been classified as pathogenic. Further analysis based on family relationships confirmed the maternal origin of the c.183_184delTAinsCT mutation and the paternal origin of the c.258 + 2 T > C mutation. Somatic mutations in SAMD9L, SETBP1, BCL11B, and RNASEL were also detected. Based on these findings, a diagnosis of Shwachman Diamond syndrome (SDS) was made. Subsequently, we initiated the search for a suitable unrelated donor (UD) due to the patient’s frequent transfusion requirements for red RBC and PLTs. Interestingly, the patient’s condition gradually improved after 3 months of diagnosis, leading us to adopt a watch-and-wait strategy given the stable disease course. Eighteen months after the initial diagnosis, the child is free from transfusion but CR has not been achieved.

Case 3 (N10) was a 13-year-old boy who presented with dizziness, anorexia, and fatigue for 4 days. Laboratory results revealed pancytopenia with Hb levels of 5.7 g/ dl, PLTs of 15 × 10^9^/L, and ANC of 2 × 10^9^/L. Further cytogenetic examination of 20 metaphases exhibited monosomy 7 (confirmed to be of somatic origin). WES detected somatic GATA2 and RUNX1 mutations along with a de novo pathogenic heterozygous variant in GATA2 (c.917 G>A(p.Trp306Ter). HLA typing of the children and his family member was initiated immediately after a diagnosis of MDS-EB was confirmed. A search for a suitable UD was then started for the lack of HLA-identical siblings. However, due to irregular follow-up, a BM aspirate repeated 2 months later showed 25% blasts with multilineage dysplasia and a positive EVI1 fusion gene, suggesting progression to AML. The child achieved CR after multiple cycles of cytoreductive chemotherapy and received HSCT from a sibling donor (6/10 HLA loci) as a fully matched UD could not be found. Unfortunately, the patient succumbed to a severe infection 2 months after HSCT.

## Discussion

In the last decade, advancements in DNA sequencing technologies have contributed to a better understanding of the genetic factors involved in childhood MDS. Growing evidence suggests that childhood MDS may be associated with germline susceptibility [4, 14, 15]. Patients with germline syndromes caused by variants in GATA2, ETV6, RUNX1, SAMD9/SAMD9-L and SRP72 have been shown to be susceptible to MDS. However, the mechanisms through which germline susceptibility leads to MDS are not fully understood. A few studies [7, 16, 17] indicated that somatic mutations played a major part in the pathogenesis of germline MDS. The acquisition of additional somatic mutations may be necessary for MDS initiation. Driving somatic mutations occurs on the background of a germline lesion that is responsible for a faster mutational rate in hematopoietic cells or selective pressure driving clonal outgrowth [5, 6]. In addition, there are certain recurrent chromosomal aberrations observed in partial germline MDS. For example, monosomy 7 is common in patients with germline SAMD9/9L and GATA2 mutations [18–20], while isochromosome 7q and del20q often occur in SDS [21]. The precise role of these recurrent chromosomal abnormalities in the pathogenesis of germline MDS is not fully understood. Pastor et al. [22] have revealed synergistic effects of the co-occurrence of genetic alteration events in the evolution of childhood MDS, suggesting that recurrent chromosomal abnormalities may contribute to a final common pathway of disease progression [2].

The above analysis details three cases that exhibited germline and somatic mutations, suggesting that their occurrence may result from a combination of the above mechanisms. The diversity of three cases clinical features and outcomes may be explained by oncogenic transformation or hematopoietic stem cell adaptation (improved hematopoiesis) triggered by somatic mutations driven by selective pressures on the background of germline lesions [5]. Meanwhile, these mechanisms provide strong evidence for further targeted and precise therapy. Case 2 with germline SBDS variants and somatic mutations such as del20q had a reversed disease outcome and a stable clinical course. We hypothesized the reversion of the disease outcome might be correlated with del20q. The gene encoding for eukaryotic Initiation Factor 6 (EIF6) is located on the 20q [23, 24]. Through a-CGH, Valli et al. discovered in their study that 6 SBDS patients with del20q all had EIF6 deletion, and the deletion of 20q may cause EIF6 deletion, resulting in haploinsufficiency [23, 24]. This probably brings a reduced dose of EIF6 in SBDS-deficient HSPCs, which may help bypass the restraint on growth by ameliorating the defect in ribosomal subunit joining, in turn favoring the formation of actively translating 80 S ribosomes and provide a clonal fitness advantage [23, 25–28]. This mechanism probably underlie the stable clinical course of case 2 and exploring this mechanism may provide valuable insights for treating other SDS patients without del20q. Case 3 with a germline GATA2 variant and somatic mutations including monosomy 7 and RUNX1 had a rapidly progressive course of disease. We consider this rapidly progressive clinical course may be attributed to the inability to reverse the hematopoietic defect due to multiple somatic mutation driven by selective pressures in the GATA2 germline genetic context. Case 1, on the other hand, carried germline HSP90AA1 and NUP98 variants, as well as a somatic TRAF2 mutation. She initially exhibited a stable clinical course, but her disease gradually progressed to TD. The precise role of these mutations in the disease remains unclear. This phenomenon highlights a common challenge in our understanding of the pathogenicity of genetic variants, as it tends to lag considerably behind the identification of these variants. To address this issue, it is crucial to conduct more functional testing in the future to match the rate of variant discovery in clinical sphere with robust functional data, therefore enabling the identification of new pathogenic mutations [29].

An exhaustive analysis of these 3 cases and a summary of the genetic data of 13 patients demonstrate that the clinical presentation, disease evolution and outcome of childhood MDS are closely connected with the genetic background. We found that advanced MDS, PD, and TD were more common in patients with CGSMs compared to those with somatic mutations alone or without any mutations. Additionally, we observed significant heterogeneity in clinical outcomes of the 8 CSGMs patients (4 advanced MDS and 4 RCC). The 4 advanced MDS patients had poor clinical manifestations and outcomes, with the somatic mutations all associated with MDS and the germline mutations mostly being pathogenic. The 4 RCC patients had better overall prognoses with most germline and somatic mutations of unknown significance. Our research results indicate that a patient’s genetic background is closely related to its clinical outcomes, and CSGMs contribute to disease progression and adverse clinical manifestations. Furthermore, the co-occurrence of germline pathogenic variants and MDS-associated somatic mutations may be indicative of a poor prognosis. It is important to note, however, these conclusions should be interpreted with caution due to the limitations of our sample size, and further data are required to validate this theory. In addition, our early genetic testing for pediatric MDS primarily involved targeted gene sequencing, resulting in less comprehensive detection of genetic variants in childhood MDS. Therefore, it is possible that there are other genetic factors yet to be uncovered, warranting further investigation and exploration.

An alternate strategy of genetic background guiding treatment may be included in the clinical therapeutic spectrum of pediatric MDS in the future based on our findings that clinical manifestations are closely linked with specific genetic contexts. However, the current treatment strategy of childhood MDS still depends on its distinct clinical manifestations [1, 30, 31]. The three cases elaborated in this research are used as typical examples. Both case 1 and case 2 were RCC without complex karyotype and monosomy 7. In case 1, we used a careful watch-and-wait strategy due to the absence of TD and marked neutropenia(<0.5 × 10^9^/L) at the early stage of disease. Therapeutic intervention was initiated when the child progressed to TD for PLTs. While awaiting a suitable HLA-matched UD, the patient was treated with CsA due to hypocellularity and the absence of poor-risk karyotype [1, 32, 33], but no significant improvement in the hematological parameters was observed before receiving HSCT. In case 2, HLA typing was performed as soon as the diagnosis of MDS was established due to TD. The patient gradually had a stable clinical course free from TD while awaiting a suitable UD, and ultimately leading us to continue with a watch-and-wait strategy. Consequently, for the treatment of pediatric RCC, disease status at different stages should be carefully evaluated to develop the most appropriate treatment strategy. HSCT represents the therapy of choice for advanced MDS in children [34, 35]. For case 3, HLA matching was initiated shortly after the diagnosis was made and cytoreductive chemotherapy was administered before HSCT due to the rapid progress of his disease into AML within two months. Unfortunately, prognosis for this patient was unfavorable because the patient was older than 12 years at the time of HSCT and a longer interval of more than 4 months between the diagnosis and HSCT. Therefore, close monitoring of BM status is essential for patients with advanced pediatric MDS to detect any disease progression promptly, ensuring HSCT is performed as soon as possible.

## Conclusion

The exploration of genetic factors in childhood MDS and their impact on prognosis has shed light on the developmental mechanisms underlying the disease and their correlation with disease characteristics and outcomes. However, further studies are required to validate these findings. Future advancements in the understanding of MDS genetic factors hold the potential to aid in risk stratification, identify therapeutic targets, guide clinical decisions-making, and customize individualized therapeutic approaches.

### Electronic supplementary material

Below is the link to the electronic supplementary material.

Supplementary Material 1

## Data Availability

Partial genomic data have been deposited in the National Center for Biotechnology Information (NCBI), under accession SRP405932. All other remaining data are available within the article, further inquiries can be directed to the corresponding author.

## References

[1] Locatelli F, Strahm B (2018). How I treat myelodysplastic syndromes of childhood. Blood.

[2] Hasle H (2016). Myelodysplastic and myeloproliferative disorders of childhood. Hematol Am Soc Hematol Educ Program.

[3] Godley LA, Shimamura A (2017). Genetic predisposition to hematologic malignancies: management and surveillance. Blood.

[4] Furutani E, Shimamura A (2017). Germline genetic predisposition to Hematologic Malignancy. J Clin Oncol.

[5] Kennedy AL, Shimamura A (2019). Genetic predisposition to MDS: clinical features and clonal evolution. Blood.

[6] Cazzola M (2020). Myelodysplastic syndromes. N Engl J Med.

[7] Schratz KE, DeZern AE (2020). Genetic predisposition to Myelodysplastic Syndrome in Clinical Practice. Hematol Oncol Clin North Am.

[8] Lindsley RC, Saber W, Mar BG, Redd R, Wang T, Haagenson MD, Grauman PV, Hu Z-H, Spellman SR, Lee SJ (2017). Prognostic mutations in Myelodysplastic Syndrome after stem-cell transplantation. N Engl J Med.

[9] Makishima H, Yoshizato T, Yoshida K, Sekeres MA, Radivoyevitch T, Suzuki H, Przychodzen B, Nagata Y, Meggendorfer M, Sanada M (2017). Dynamics of clonal evolution in myelodysplastic syndromes. Nat Genet.

[10] Arber DA, Orazi A, Hasserjian R, Thiele J, Borowitz MJ, Le Beau MM, Bloomfield CD, Cazzola M, Vardiman JW (2016). The 2016 revision to the World Health Organization classification of myeloid Neoplasms and acute Leukemia. Blood.

[11] Kim N, Pavletic S, Norsworthy KJ (2022). Meaningful response criteria for myelodysplastic syndromes. Br J Haematol.

[12] Cheson BD, Greenberg PL, Bennett JM, Lowenberg B, Wijermans PW, Nimer SD, Pinto A, Beran M, de Witte TM, Stone RM (2006). Clinical application and proposal for modification of the International Working Group (IWG) response criteria in myelodysplasia. Blood.

[13] Platzbecker U, Fenaux P, Adès L, Giagounidis A, Santini V, van de Loosdrecht AA, Bowen D, de Witte T, Garcia-Manero G, Hellström-Lindberg E (2019). Proposals for revised IWG 2018 hematological response criteria in patients with MDS included in clinical trials. Blood.

[14] Bannon SA, DiNardo CD. Hereditary predispositions to Myelodysplastic Syndrome. Int J Mol Sci 2016, 17(6). 10.3390/ijms17060838 PMC4926372 27248996

[15] Babushok DV, Bessler M (2015). Genetic predisposition syndromes: when should they be considered in the work-up of MDS?. Best Pract Res Clin Haematol.

[16] Churpek JE, Pyrtel K, Kanchi K-L, Shao J, Koboldt D, Miller CA, Shen D, Fulton R, O’Laughlin M, Fronick C (2015). Genomic analysis of germ line and somatic variants in familial myelodysplasia/acute Myeloid Leukemia. Blood.

[17] McReynolds LJ, Yang Y, Yuen Wong H, Tang J, Zhang Y, Mulé MP, Daub J, Palmer C, Foruraghi L, Liu Q (2019). MDS-associated mutations in germline GATA2 mutated patients with hematologic manifestations. Leuk Res.

[18] Yoshida M, Tanase-Nakao K, Shima H, Shirai R, Yoshida K, Osumi T, Deguchi T, Mori M, Arakawa Y, Takagi M (2020). Prevalence of germline GATA2 and SAMD9/9L variants in paediatric haematological disorders with monosomy 7. Br J Haematol.

[19] Wlodarski MW, Sahoo SS, Niemeyer CM (2018). Monosomy 7 in Pediatric Myelodysplastic syndromes. Hematol Oncol Clin North Am.

[20] Wlodarski MW, Hirabayashi S, Pastor V, Starý J, Hasle H, Masetti R, Dworzak M, Schmugge M, van den Heuvel-Eibrink M, Ussowicz M et al. Prevalence, clinical characteristics, and prognosis of GATA2-related myelodysplastic syndromes in children and adolescents. Blood 2016, 127(11). 10.1182/blood-2015-09-669937 26702063

[21] Maserati E, Minelli A, Pressato B, Valli R, Crescenzi B, Stefanelli M, Menna G, Sainati L, Poli F, Panarello C (2006). Shwachman syndrome as mutator phenotype responsible for myeloid dysplasia/neoplasia through karyotype instability and chromosomes 7 and 20 anomalies. Genes Chromosomes Cancer.

[22] Pastor V, Hirabayashi S, Karow A, Wehrle J, Kozyra EJ, Nienhold R, Ruzaike G, Lebrecht D, Yoshimi A, Niewisch M (2017). Mutational landscape in children with myelodysplastic syndromes is distinct from adults: specific somatic drivers and novel germline variants. Leukemia.

[23] Valli R, Minelli A, Galbiati M, D’Amico G, Frattini A, Montalbano G, Khan AW, Porta G, Millefanti G, Olivieri C (2019). Shwachman-Diamond syndrome with clonal interstitial deletion of the long arm of chromosome 20 in bone marrow: haematological features, prognosis and genomic instability. Br J Haematol.

[24] Valli R, Pressato B, Marletta C, Mare L, Montalbano G, Curto FL, Pasquali F, Maserati E (2013). Different loss of material in recurrent chromosome 20 interstitial deletions in Shwachman-Diamond syndrome and in myeloid Neoplasms. Mol Cytogenet.

[25] Kennedy AL, Myers KC, Bowman J, Gibson CJ, Camarda ND, Furutani E, Muscato GM, Klein RH, Ballotti K, Liu S (2021). Distinct genetic pathways define pre-malignant versus compensatory clonal hematopoiesis in Shwachman-Diamond syndrome. Nat Commun.

[26] Jaako P, Faille A, Tan S, Wong CC, Escudero-Urquijo N, Castro-Hartmann P, Wright P, Hilcenko C, Adams DJ, Warren AJ (2022). eIF6 rebinding dynamically couples ribosome maturation and translation. Nat Commun.

[27] Warren AJ (2018). Molecular basis of the human ribosomopathy Shwachman-Diamond syndrome. Adv Biol Regul.

[28] Tan S, Kermasson L, Hilcenko C, Kargas V, Traynor D, Boukerrou AZ, Escudero-Urquijo N, Faille A, Bertrand A, Rossmann M (2021). Somatic genetic rescue of a germline ribosome assembly defect. Nat Commun.

[29] Klco JM, Mullighan CG (2021). Advances in germline predisposition to acute leukaemias and myeloid Neoplasms. Nat Rev Cancer.

[30] Kardos G, Baumann I, Passmore SJ, Locatelli F, Hasle H, Schultz KR, Starý J, Schmitt-Graeff A, Fischer A, Harbott J (2003). Refractory anemia in childhood: a retrospective analysis of 67 patients with particular reference to monosomy 7. Blood.

[31] Göhring G, Michalova K, Beverloo HB, Betts D, Harbott J, Haas OA, Kerndrup G, Sainati L, Bergstraesser E, Hasle H (2010). Complex karyotype newly defined: the strongest prognostic factor in advanced childhood Myelodysplastic Syndrome. Blood.

[32] Yoshimi A, Baumann I, Führer M, Bergsträsser E, Göbel U, Sykora K-W, Klingebiel T, Gross-Wieltsch U, van den Heuvel-Eibrink MM, Fischer A (2007). Immunosuppressive therapy with anti-thymocyte globulin and cyclosporine A in selected children with hypoplastic refractory cytopenia. Haematologica.

[33] Sloand EM, Wu CO, Greenberg P, Young N, Barrett J (2008). Factors affecting response and survival in patients with myelodysplasia treated with immunosuppressive therapy. J Clin Oncol.

[34] Galaverna F, Ruggeri A, Locatelli F (2018). Myelodysplastic syndromes in children. Curr Opin Oncol.

[35] Strahm B, Nöllke P, Zecca M, Korthof ET, Bierings M, Furlan I, Sedlacek P, Chybicka A, Schmugge M, Bordon V (2011). Hematopoietic stem cell transplantation for advanced Myelodysplastic Syndrome in children: results of the EWOG-MDS 98 study. Leukemia.

