# Supplementary material for: The role of genetic factors in pediatric myelodysplastic syndromes with different outcomes
Source: BMC Pediatr. 2024 Jan 8;24:28. doi: 10.1186/s12887-023-04492-2 (PMC10773107; doi:10.1186/s12887-023-04492-2)
Supplement: Supplementary file 1 — Supplementary Material 1 [file 12887_2023_4492_MOESM1_ESM.docx]

**Supplementary Table 1. Genes analyzed using targeted NGS panel (67)**

| Gene | RefSeq Accession | Gene | RefSeq Accession |
| --- | --- | --- | --- |
| ABL1 | [NM 005157](https://www.ncbi.nlm.nih.gov/nuccore/NM_005157.6/) | NF1 | [NM 001042492](https://www.ncbi.nlm.nih.gov/nuccore/NM_001042492.3/) |
| ANKRD26 | [NM 014915](https://www.ncbi.nlm.nih.gov/nuccore/NM_014915.3/) | NFE2 | [NM 001136023](https://www.ncbi.nlm.nih.gov/nuccore/NM_001136023.3/) |
| ASXL1 | [NM 015338](https://www.ncbi.nlm.nih.gov/nuccore/NM_015338.6/) | NOTCH1 | [NM 017617](https://www.ncbi.nlm.nih.gov/nuccore/NM_017617.5/) |
| ATM | [NM 000051](https://www.ncbi.nlm.nih.gov/nuccore/NM_000051.4/) | NPM1 | [NM 002520](https://www.ncbi.nlm.nih.gov/nuccore/NM_002520.7/) |
| BCOR | [NM 001123385](https://www.ncbi.nlm.nih.gov/nuccore/NM_001123385.2/) | NRAS | [NM 002524](https://www.ncbi.nlm.nih.gov/nuccore/NM_002524.5/) |
| BCORL1 | [NM 021946](https://www.ncbi.nlm.nih.gov/nuccore/NM_021946.5/) | PDGFRA | [NM 006206](https://www.ncbi.nlm.nih.gov/nuccore/NM_006206.6/) |
| BRAF | [NM 004333](https://www.ncbi.nlm.nih.gov/nuccore/NM_004333.6/) | PHF6 | [NM 032458](https://www.ncbi.nlm.nih.gov/nuccore/NM_032458.3/) |
| CALR | [NM 004343](https://www.ncbi.nlm.nih.gov/nuccore/NM_004343.4/) | PIGA | [NM 002641](https://www.ncbi.nlm.nih.gov/nuccore/NM_002641.4/) |
| CBL | [NM 005188](https://www.ncbi.nlm.nih.gov/nuccore/NM_005188.4/) | PRPF40B | [NM 001031698](https://www.ncbi.nlm.nih.gov/nuccore/NM_001031698.3/) |
| CEBPA | [NM 004364](https://www.ncbi.nlm.nih.gov/nuccore/NM_004364.5/) | PRPF8 | [NM 006445](https://www.ncbi.nlm.nih.gov/nuccore/NM_006445.4/) |
| KIT | [NM 000222](https://www.ncbi.nlm.nih.gov/nuccore/NM_000222.3/) | PTEN | [NM 000314](https://www.ncbi.nlm.nih.gov/nuccore/NM_000314.8/) |
| CSF3R | [NM 156039](https://www.ncbi.nlm.nih.gov/nuccore/NM_156039.3/) | PTPN11 | [NM 002834](https://www.ncbi.nlm.nih.gov/nuccore/NM_002834.5/) |
| CSMD1 | [NM 033225](https://www.ncbi.nlm.nih.gov/nuccore/NM_033225.6/) | RAD21 | [NM 006265](https://www.ncbi.nlm.nih.gov/nuccore/NM_006265.3/) |
| CUX1 | [NM 001202543](https://www.ncbi.nlm.nih.gov/gene/?term=NM+001202543) | ROBO1 | [NM 002941](https://www.ncbi.nlm.nih.gov/nuccore/NM_002941.4/) |
| DDX41 | [NM 016222](https://www.ncbi.nlm.nih.gov/nuccore/NM_016222.4/) | ROBO2 | [NM 001128929](https://www.ncbi.nlm.nih.gov/nuccore/NM_001128929.3/) |
| DNMT3A | [NM 022552](https://www.ncbi.nlm.nih.gov/nuccore/NM_022552.5/) | RUNX1 | [NM 001754](https://www.ncbi.nlm.nih.gov/nuccore/NM_001754.5/) |
| EP300 | [NM 001429](https://www.ncbi.nlm.nih.gov/nuccore/NM_001429.4/) | SETBP1 | [NM 015559](https://www.ncbi.nlm.nih.gov/nuccore/NM_015559.3/) |
| ETNK1 | [NM 018638](https://www.ncbi.nlm.nih.gov/nuccore/NM_018638.5/) | SF1 | [NM 001178030](https://www.ncbi.nlm.nih.gov/nuccore/NM_001178030.2/) |
| ETV6 | [NM 001987](https://www.ncbi.nlm.nih.gov/nuccore/NM_001987.5/) | SF3A1 | [NM 005877](https://www.ncbi.nlm.nih.gov/nuccore/NM_005877.6/) |
| EZH2 | [NM 004456](https://www.ncbi.nlm.nih.gov/nuccore/NM_004456.5/) | SF3B1 | [NM 012433](https://www.ncbi.nlm.nih.gov/nuccore/NM_012433.4/) |
| FLT3 | [NM 004119](https://www.ncbi.nlm.nih.gov/nuccore/NM_004119.3/) | SH2B3 | [NM 005475](https://www.ncbi.nlm.nih.gov/nuccore/NM_005475.3) |
| GATA1 | [NM 002049](https://www.ncbi.nlm.nih.gov/nuccore/NM_002049.4/) | SMC1A | [NM 006306](https://www.ncbi.nlm.nih.gov/nuccore/NM_006306.4/) |
| GATA2 | [NM 032638](https://www.ncbi.nlm.nih.gov/nuccore/NM_032638.5/) | SMC3 | [NM 005445](https://www.ncbi.nlm.nih.gov/nuccore/NM_005445.4/) |
| GNAS | [NM 080425](https://www.ncbi.nlm.nih.gov/nuccore/NM_080425.4/) | SRP72 | [NM 006947](https://www.ncbi.nlm.nih.gov/nuccore/NM_006947.4/) |
| IDH1 | [NM 005896](https://www.ncbi.nlm.nih.gov/nuccore/NM_005896.4/) | SRSF2 | [NM 001195427](https://www.ncbi.nlm.nih.gov/gene/?term=NM+001195427) |
| IDH2 | [NM 002168](https://www.ncbi.nlm.nih.gov/nuccore/NM_002168.4/) | STAG2 | [NM 001042749](https://www.ncbi.nlm.nih.gov/nuccore/NM_001042749.2/) |
| IKZF1 | [NM 006060](https://www.ncbi.nlm.nih.gov/nuccore/NM_006060.6/) | SUZ12 | [NM 015355](https://www.ncbi.nlm.nih.gov/nuccore/NM_015355.4/) |
| JAK1 | [NM 002227](https://www.ncbi.nlm.nih.gov/nuccore/NM_002227.4/) | TET2 | [NM 001127208](https://www.ncbi.nlm.nih.gov/nuccore/NM_001127208.3/) |
| JAK2 | [NM 004972](https://www.ncbi.nlm.nih.gov/nuccore/NM_004972.4/) | TP53 | [NM 000546](https://www.ncbi.nlm.nih.gov/nuccore/NM_000546.6/) |
| JAK3 | [NM 000215](https://www.ncbi.nlm.nih.gov/nuccore/NM_000215.4/) | U2AF1 | [NM 006758](https://www.ncbi.nlm.nih.gov/nuccore/NM_006758.3/) |
| KMT2C | [NM 170606](https://www.ncbi.nlm.nih.gov/nuccore/NM_170606.3/) | U2AF2 | [NM 007279](https://www.ncbi.nlm.nih.gov/nuccore/NM_007279.3/) |
| KMT2D | [NM 003482](https://www.ncbi.nlm.nih.gov/nuccore/NM_003482.4/) | WT1 | [NM 024426](https://www.ncbi.nlm.nih.gov/gene/?term=NM+024426) |
| KRAS | [NM 033360](https://www.ncbi.nlm.nih.gov/nuccore/NM_033360.4/) | ZRSR2 | [NM 005089](https://www.ncbi.nlm.nih.gov/nuccore/NM_005089.4/) |
| MPL | [NM 005373](https://www.ncbi.nlm.nih.gov/nuccore/NM_005373.3/) |  |  |
